# Quantum dots based in-vitro co-culture cancer model for identification of rare cancer cell heterogeneity

**DOI:** 10.1038/s41598-022-09702-y

**Published:** 2022-04-07

**Authors:** Satyanarayana Swamy Vyshnava, Gayathri Pandluru, Kanderi Dileep Kumar, Shiva Prasad Panjala, Swathi Banapuram, Kameshpandian Paramasivam, Kothamunireddy Varalakshmi Devi, Roja Rani Anupalli, Muralidhara Rao Dowlatabad

**Affiliations:** 1 Department of Biotechnology, University College of Sciences, Sri Krishnadevaraya University, Anantapuramu, Andhra Pradesh 515003 India; 2 Department of Microbiology, University College of Sciences, Sri Krishnadevaraya University, Anantapuramu, Andhra Pradesh 515003 India; 3 Department of Genetics and Biotechnology, University College of Sciences, Osmania University, Hyderabad, Telangana 500007 India; 4 Department of Forensic Science, Ultra Arts and Science College, Madurai, Tamil Nādu 625104 India; 5 College of Pharmaceutical Sciences, Sri Krishnadevaraya University, Anantapuramu, Andhra Pradesh 515003 India

**Keywords:** Cancer imaging, Cancer models, Cancer screening, Metastasis, Tumour heterogeneity, Nanobiotechnology, Nanoscale materials, Biochemistry, Biotechnology, Cancer, Cell biology, Chemical biology, Immunology, Chemistry, Materials science, Nanoscience and technology

## Abstract

Cancer cell heterogeneity (CCH) is crucial in understanding cancer progression and metastasis. The CCH is one of the stumbling blocks in modern medicine's therapeutics and diagnostics . An in-vitro model of co-culture systems of MCF-7, HeLa, HEK-293, with THP-1 cells showed the occurrence of EpCAM positive (EpCAM+) and EpCAM negative (EpCAM−) heterogenetic cancer cell types labeled with the Quantum Dot antibody conjugates (QD^Ab^). This in-vitro model study could provide insights into the role of rare cancer cells manifestation and their heterogeneity in metastatic progression and risk for severe infections in these patients. We successfully report the presence of CCH based on the fluorescence ratios of the co-cultured cancer cells when treated with the QD^Ab^. These short-term mimic co-cultures give a compelling and quite associated model for assessing early treatment responses in various cancers.

## Introduction

Metastatic cancers are the leading cause of mortality due to poor prognosis and early progressive disease^1,2^. Earlier studies suggested that CCH subsists in metastatic stages that invade the bloodstream after being disseminated from the primary site of infection^3,4^. Tumor cell heterogeneity and tumor morphologic heterogeneity, which is at the heart of many tumor grading and prognostic classification systems, has recently gained more attention^5^. Cell proliferation, immune infiltration, differentiation status, and necrosis can differ between microscopic fields within a tumor due to its heterogeneity^4,6^. Intra-tumor heterogeneity and inter-tumor heterogeneity, among other molecular, phenotypic, and functional characteristics, can obstruct diagnosis and pose therapeutic challenges in bone^7,8^, lung^9,10^, brain^11,12^, and liver^13,14^ cancer metastasis; suggesting that there is currently no systematic and comprehensive assessment of the molecular makeup for metastasis^15^.

During metastasis cancer cells invade to distant sites mainly through circulating blood, often executing a cascade of intravasation-translocation-extravasation-colonization to overcome the physical constraints of properties like adherence and stroma, which act as barriers^16,17^. The cancer cells may undergo transitions to enhance their motility and extravasate to colonize at distant locations^1,18^. Prior studies have demonstrated the existence of phenotypically different subpopulations of tumorigenic and non-tumorigenic cells in various human malignancies, including acute myeloid leukaemia^19,20^, chronic myeloid leukaemia^21,22^, breast cancer^23,24^, glioblastoma^25,26^, colorectal cancer^27,28^, pancreatic cancer^29,30^, and ovarian cancers^23,31^. Recent investigations revealed the presence of multiple heterogenic cancer cells, such as Epithelial to mesenchymal transition (EMTs)^32,33^, Mesenchymal to epithelial transitions (METs)^18,34^, Circulating tumor cells (CTCs)^24,35^, Disseminated Tumor Cells (DTCs)^36,37^, etc., in many cancer progression and metastasis.

These EMT and MET are to be considered as one of the significant reasons for CCH, implicating the biological characteristics of individual CTCs in the blood circulation, in addition to their number^38^. Despite this, most tumor cells die during transit due to biological and physical constraints such as shear stress and immune surveillance (apoptosis and anoikis), and only a small subset of surviving CTCs (approximately 0.01%) develop tumor-initiating cell potential^38,39^. CTCs are involved in distant metastasis along with the drug sensitivity and apoptosis resistance^12,24,40^.

The molecular basis of CCH started with enriched fractions, which made limited information on tumor heterogeneity^3,41^. Due to recent advances in single-cell technologies, CCH-specific genetic mutations have been discovered, and stereotyping of the CCH population has revealed the emergence of subclones with vibrant phenotypic traits. Contributing significantly to the evolution of the tumor genome during disease progression and treatment^38,40^. The clinical significance of the CCH such as CTC’s were detected with the CellSearch® system, which removes only the majority of leukocytes by immunomagnetic enrichment of cells using epithelial cell adhesion molecule (EpCAM) antibodies coupled to ferrofluids, which lack immunological marker like CD45^42^. Similar to the CellSearch® system there are very limited technologies existing currently which can effectively isolate a restricted number of CCH cell types from metastasis patients based on the molecular markers using nanotechnological applications^43–45^. In the present study, we isolated the heterogenic tumor cells with morphological characteristics such as the presence of a nucleus and the absence of certain cell surface markers in a co-culture system of breast, cervical and renal cancer cell lines with monocytes through an in-vitro approach using a Quantum dot antibody conjugate platform.

## Methods

### Materials

Chemicals listed were purchased include Cadmium Oxide-99% (CdO), Zinc Acetate-99% (Zn(Act)2), Oleic Acid (OA), Sulfur powder-99% (S), Diphenylphosphine 98% (DPP), Trioctylphosphine 97% (TOP), Tetra-methyl-ammonium-hydroxide Solution (TMAH), N-hydroxy-succinimide-98% (NHS), and NH_2_-(PEG)_8_-Propionic acid from Sigma-Aldrich. 1-Octadecene-90% (ODE) from Acros Organics. Chloroform, Acetone, Methanol, and Isopropanol (ISP) from J.T.Baker. 3-Mercaptopropionic acid ≥ 99% (MPA) from Merckmillipore. 1-ethyl-3-[3-dimethylaminopropyl] carbodiimide-98% (EDC) from Tokyo Chemical Industry Co. And Streptavidin (SA) from Alfa Aesar, Cell culture reagents include Dulbecco’s modified Eagle’s Medium (DMEM), Roswell Park Memorial Institute-1640 medium (RPMI-1640), Fetal bovine serum (FBS), 1 mg/mL glutamine, and 100 μg/mL Streptomycin/Penicillin solution, from Gibco Bio Sciences. 3-(4,5-Dimethylthiazol-2-yl)-2,5-Diphenyl tetrazolium Bromide (MTT) and Sodium lauryl sulfate (SDS) from thermo scientific and other related chemicals are further mentioned. .

### Quantum dots synthesis

CdS/ZnS with OA as capping are prepared as described in the established protocol^46,47^ and labeled as ${QD}_{CdS/ZnS}^{450}$, CdSe/ZnS with DPP as capping agent were prepared as described with minor modifications in the synthesis process^46,48^ and labeled as ${QD}_{CdSe/ZnS}^{525}$,. The CdSe/ZnS with TOP capped QD’s were prepared as described with minor modifications in the synthesis process^49–51^ and labeled as ${QD}_{CdSe/ZnS}^{615}$.

The synthesized QD’s are characterized for the fluorescence and absorbance spectrum with UV–Visible Spectroscopy (SpectraMax®, M2e Molecular Devices). The morphological distributions are further studied using the High-resolution transmission electron microscopy (HR-TEM) (JAPAN ELECTRON OPTICS LABORATORY CO., LTD), the crystalline structure were observed using the X-ray diffraction spectroscopy (XRD) (Rigaku).

### Conjugation of antibodies to quantum dots

To conjugate, the antibodies (Abs) on the surface of QD’s, including Anti-EpCAM, Anti-CD45, and Anti-CD44, were coupled with biotin. (Thermofisher Scientific Inc. and Sigma Aldrich). All the Abs are stored at − 20 °C to extend their shelf-life. QD’s are primarily modified with MPA through biphasic ligand exchange to attain active –COO^-^ group on the surface of QD’s. These QD^MPA^ are allowed for modification with NH_2_-(PEG)_8_-COOH (PEG) and finally conjugated with SA molecules with EDC/NHS coupling chemistry as mentioned in the earlier reports^46^. The conjugation of PEG and SA on the surface of QDs was successfully achieved by adding an equimolar mixture of 1 mM EDC and NHS which was prepared in the MES (2-(N-morpholino) ethane sulfonic acid) buffer at 4 °C (all the reactions are to be worked under the chilling condition to maintain the integrity of the PEG and SA). 1 mg of the individual QD^MPA^ are measured and dissolved in Millipore grade water, followed by the addition of the 200 μL of EDC. The reaction was incubated at 37 °C for 15–30 min followed by the addition of 200 μL of NHS for the next 15 min to form an unstable intermediate compound of active carboxylic group facilitating the formation of peptide bond with PEG. 1 μg/lit of SA was added to the above solution and allowed to develop a strong QD^MPA/PEG/SA^. The QD^MPA/PEG/SA^ were subjected to dialysis to remove excess PEG and SA. The purified QD^MPA/PEG/SA^ react with Abs-biotin moiety to form a strong biotin-streptavidin binding. To define the specific Abs, QD^450^ was bound with Anti-EpCAM (${QD}_{CdS/ZnS}^{450/MPA/PEG/SA/EpCAM}$), QD^525^ with Anti-CD45 (${QD}_{CdSe/ZnS}^{525/MPA/PEG/SA/CD45}$), and QD^615^ with Anti-CD44(${QD}_{CdSe/ZnS}^{615/MPA/PEG/SA/CD44}$). These QDs were subjected to purification by simple dialysis via dialysis cassettes (Thermo scientific Inc.). Later all the QDs with Ab are stored under freezing conditions to enhance the shelf life of the QD^Ab^.

### Cell culture and cytotoxicity

The MCF-7 (Human breast cancer cell line) HeLa (Human cervical cancer cell line) cell lines are acquired from the National Centre for Cell Science (NCCS, Pune, India). Wherein, HEK-293 (Human embryonic kidney cell line) and THP-1(Human monocytic cell line) are brought from Bioresource Collection and Research Center (BCRC, Hsinchu, Taiwan). These cell lines were cultured in DMEM except the THP-1 grown in RPMI-1640 medium, supplemented with 10% FBS, 1 mg/mL glutamine, and 100 μg/mL Streptomycin/Penicillin solution at 37 °C in a humidified atmosphere with 5% CO_2_. The MTT and SDS stock solutions were prepared according to the manufacturer's instructions for cytotoxicity assays and stored at 4 °C, protected from light. Individual cells were seeded in the population of 1 × 10^4^ cells/mL in the 96 well flat bottom microtiter plates overnight before adding the QD^MPA^. The desired amount of individual stock QD^MPA^ with concentrations of 0.1, 1, 5,10, 25, 50, and 100 µg/mL was dissolved in the DMEM media (without phenol and FBS). 100 μl of each stock solution was added to the respective wells in the microtiter plates of the MCF-7, HeLa, HEK-293, and THP-1cells. These cells were cultured at optimal growth conditions for 24 h. The cell viability was determined by addition of 10 μl of the MTT- PBS (MTT dissolved in Phosphate buffer saline) solution, and was incubated for 4 h. Subsequently, 100 μl SDS-HCl solution was added to the respective wells to dissolve the formazan crystals formed from the viable cells. Optical absorbance was read at 570 nm to quantify the MTT that reacted with the viable cells on a multi-plate reader (Multiskan Go, Thermo Scientific).

### Co-cultures

The THP-1 cells were plated in 6-well culture dishes, and various quantities of cancer cells (MCF-7, HeLa, and HEK-293) were introduced to the THP-1 plates, as shown in the supplementary information Table S1. After 6 h, the cells were treated with predetermined concentrations of QDs specific Anti-EpCAM, Anti-CD45, and Anti-CD44 Abs coupled QDs . After 24 h of exposure, cell viability and QD-specific binding were evaluated using, confocal imaging and flow cytometry analysis to illustrate the co-culture system .

### Confocal microscopy

Compatibility of the QD^Ab^ with the HeLa, MCF-7, HEK-293 and THP-1 cell lines were observed through cellular imaging. To study the QDs uptake and coupling, the individual cell lines were allowed to seed on the coverslips at 1 × 10^6^ cells/well in a sterile 6-well plate. Later the cells were allowed to settle for 24 h, to observe the health and confluence (≈ > 60%), then celss were washed with 1 × PBS (twice or thrice). Add 1 mL of freshly prepared media dispersed with QD^Ab^ with respective concentrations (most viable cells are observed at ≈ < 5 µg/mL). The cells were incubated for 20 min, and then washed with 1 × PBS to remove excess QD^Ab^, and then add fresh 1 × PBS. The cells were observed under Confocal laser microscope (FV1000, Olympus Co.) with magnifications 10X, 40X, and 60X using the manual and automated interfaces for 405 nm laser excitations for QD^Ab^ fluorescence emissions. The RAW images are captured and processed with Olympus flow view FVW-ASW software. These cells were stored with 95% paraformaldehyde or methanol for future usage.

### Flow cytometry assay

In all experiments (we used various combinations of cell cultures designated as Trails- I, II and III), each co-culture combination was seeded onto a 12-well culture plate at 1 × 10^6^ cells/well for the flow cytometry assay as mentioned in the supplementary information Table S2. The cells were incubated for 24 h. Later the culture media was replaced, and prepared QD^Ab^ was added into the wells and incubated with an optimized protocol respectively. After incubation, the cells were rinsed with 1X PBS and allowed to settle for 5 min before treating with EDTA solution(which is comparatively less damaging to the cells than trypsin), followed by cell suspension in 1 × PBS at 4 °C. The respective QD^Ab^ binding fluorescence intensity was then evaluated using multi-laser flow cytometry and sorting assay (FACSJazz, Becton Dickinson Co).

### Statistical modelling

For statistical analysis, GraphPad Prism 5 (GraphPad Software, USA) and Origin 8.0 (OriginLab, USA) were employed. The mean, standard deviation of three independent trails is used to represent the data. One-way analysis of variance was used to establish statistical significance, followed by a t-test, where *p* < 0.05 differences were deemed to be significant.

## Results

### Characterization of the synthesized Quantum dots

**Figure 1:**
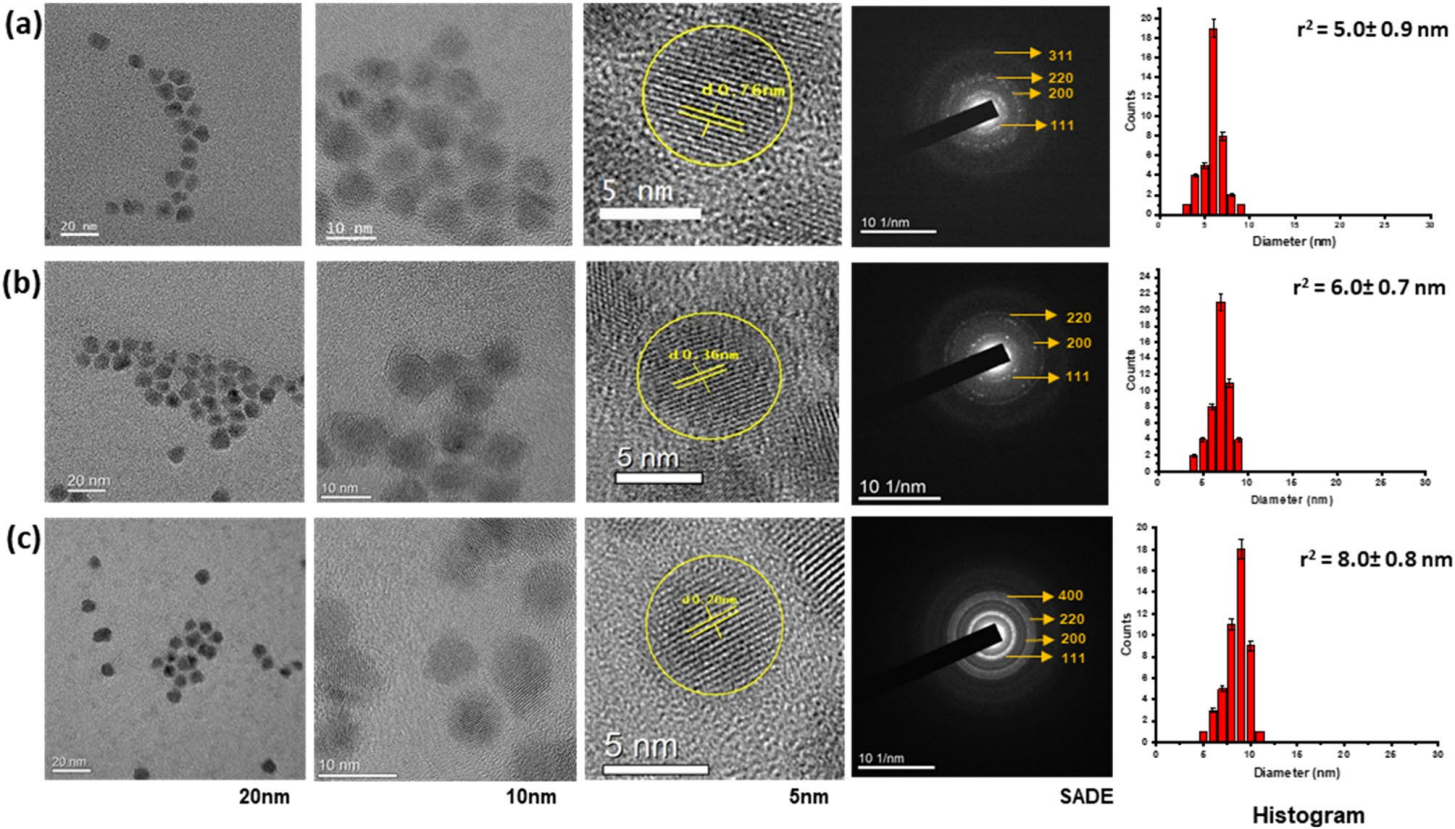
Transmission electron microscopy, selected area electron diffraction and dynamic light scattering images of Quantum dots dispersed in n-Hexanes, images are represented from left to right with enhanced resolutions from 20 to 5 nm (**a**) ${QD}_{CdS/ZnS}^{450}$ (**b**) ${QD}_{CdSe/ZnS}^{525}$ (**c**) ${QD}_{CdSe/ZnS}^{615}$.

**Figure 2:**
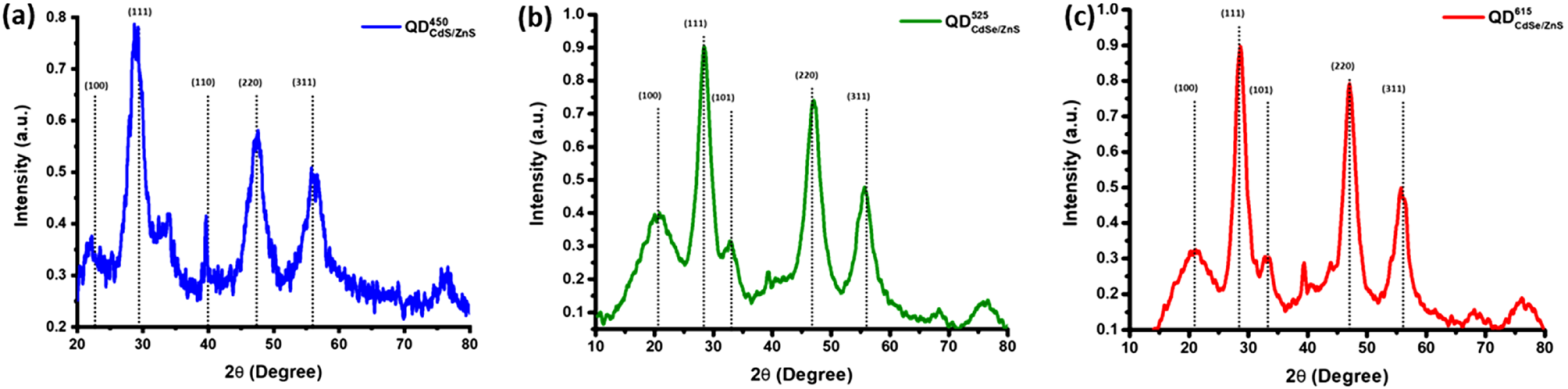
X-ray diffraction spectrum of the quantum dots in powder form (**a**) ${QD}_{CdS/ZnS}^{450}$ (**b**) ${QD}_{CdSe/ZnS}^{525}$ (**c**) ${QD}_{CdSe/ZnS}^{615}$.

**Figure 3:**
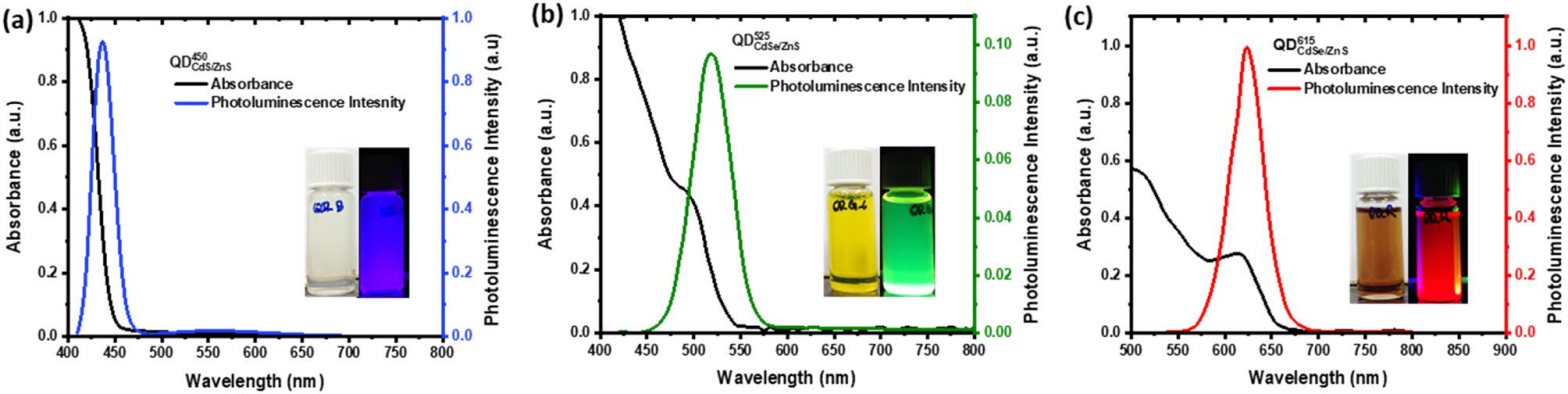
UV–Visible absorbance and fluorescence spectrum of Quantum dots dispersed in n-Hexanes (**a**) ${QD}_{CdS/ZnS}^{450}$ showing fluorescence at 450 nm and strong absorbance 410 nm, insight shows the blue fluorescence (**b**) ${QD}_{CdSe/ZnS}^{525}$ showing fluorescence at 525 nm and strong absorbance 480 nm, insight shows the green fluorescence (**c**) ${QD}_{CdSe/ZnS}^{615}$ showing fluorescence at 615 nm and strong absorbance 580 nm, insight shows the red fluorescence.

The morphology of the synthesized QD’s include ${QD}_{CdS/ZnS}^{450}$, ${QD}_{CdSe/ZnS}^{525}$, and ${QD}_{CdSe/ZnS}^{615}$ were characterized using the HR-TEM, which were shown in Fig. 1. The cores/shell size of the QDs are ${QD}_{CdS/ZnS}^{450}$ is 5.0 ± 0.9 nm, ${QD}_{CdSe/ZnS}^{525}$ is 6.0 ± 0.7 nm and ${QD}_{CdSe/ZnS}^{615}$ is 8.0 ± 0.8 nm.. The selected area electron diffraction (SAED) patterns as shown in Fig. 1 for the ${QD}_{CdS/ZnS}^{450}$, ${QD}_{CdSe/ZnS}^{525}$, and ${QD}_{CdSe/ZnS}^{615}$ revealed broad diffused circles, which indicate the synthesized QDs are in nanoscale. The diffraction patterns designated for planes of zinc blende cubic phases was (111), (200) and (220) respectively, these planes are consistent with the XRD pattern shown in Fig. 2. The XRD patterns of all the QD’s contain three primary diffraction peaks corresponding to the zinc blende cubic structure's (100), (111), (101) and (220) reflection planes (JCPDS:19-0191), which reveals that all the samples are in a single phase. The widening diffraction peaks confirms that the synthesized QDs are in nanoscale. The UV–Visible spectral analysis, as shown in Fig. 3, refers to the absorbance (AB), and the photoluminescence (PL) spectra of the QDs (${QD}_{CdS/ZnS}^{450}$, ${QD}_{CdSe/ZnS}^{525}$, and ${QD}_{CdSe/ZnS}^{615}$) which are dispersed in n-Hexanes. The respective QDs have a significant luminescence peak maxima at 450 nm, 525 nm and 615 nm, which corresponds to the blue, green and red-light emissions in the visible spectrum when excited the samples at 405 nm laser.

The QDs dissolved in n-Hexanes were subjected to biphasic ligand exchange to obtain biocompatibility and reduced cytotoxicity. An initial biphasic exchange was done using the previous reports of Vyshnava et al., 2020 and other studies^46,52,53^. The synthesized QDs were re-dispersed in the chloroform followed by the addition of monodentate hydrophilic ligand (MPA). The weak surface ligands, such as the OA, DPP and TOP on the QDs was removed during this biphasic procedure and replaced with MPA in an aqueous solution (Millipore grade water) at 37 °C. After preparation of QD^MPA^ the efficacy of the exchange process was determined primarily by ultrafiltration and subsequently concentrated by ultracentrifugation (20,000 rpm for 15 min at 4 °C) in an aqueous solution. The FTIR spectrum for the respective QD^MPA^ was consistent with the previous reports^46,48^ and the related data was shown in the supplementary information Fig. S1 for ${QD}_{CdS/ZnS}^{450/MPA}$, ${QD}_{CdSe/ZnS}^{525/MPA}$, and ${QD}_{CdSe/ZnS}^{615/MPA}$ . After ligand exchange, strong bands at 1047 cm^−1^ and 2901 cm^−1^, specific for the amide (C-N) and sulphide (-SH) groups, were observed, and the spectra were consistent with native organic ligands, including carbonyl (−C = O) characteristic stretching bands at 1242 cm^−1^ and 1646 cm^−1^, specific for MPA capped QDs. The hydroxyl (–OH) group vibration peak at 3339 cm^−1^ indicates the presence of water molecules. Large amounts of water molecules were adsorbed on the surface of CdS/CdSe QDs due to the high surface-to-volume ratio. The presence of OA, DPP and TOP is indicated by (-C-H) stretching in the band at 900 cm^−1^, 2922 cm^−1^ and 2960 cm^−1^ as shown in supplementary information Fig S1, and the absence of this peak after ligand exchange on the surface of the QDs indicates complete replacement of OA, DPP and TOP ligands with MPA.

**Figure 4:**
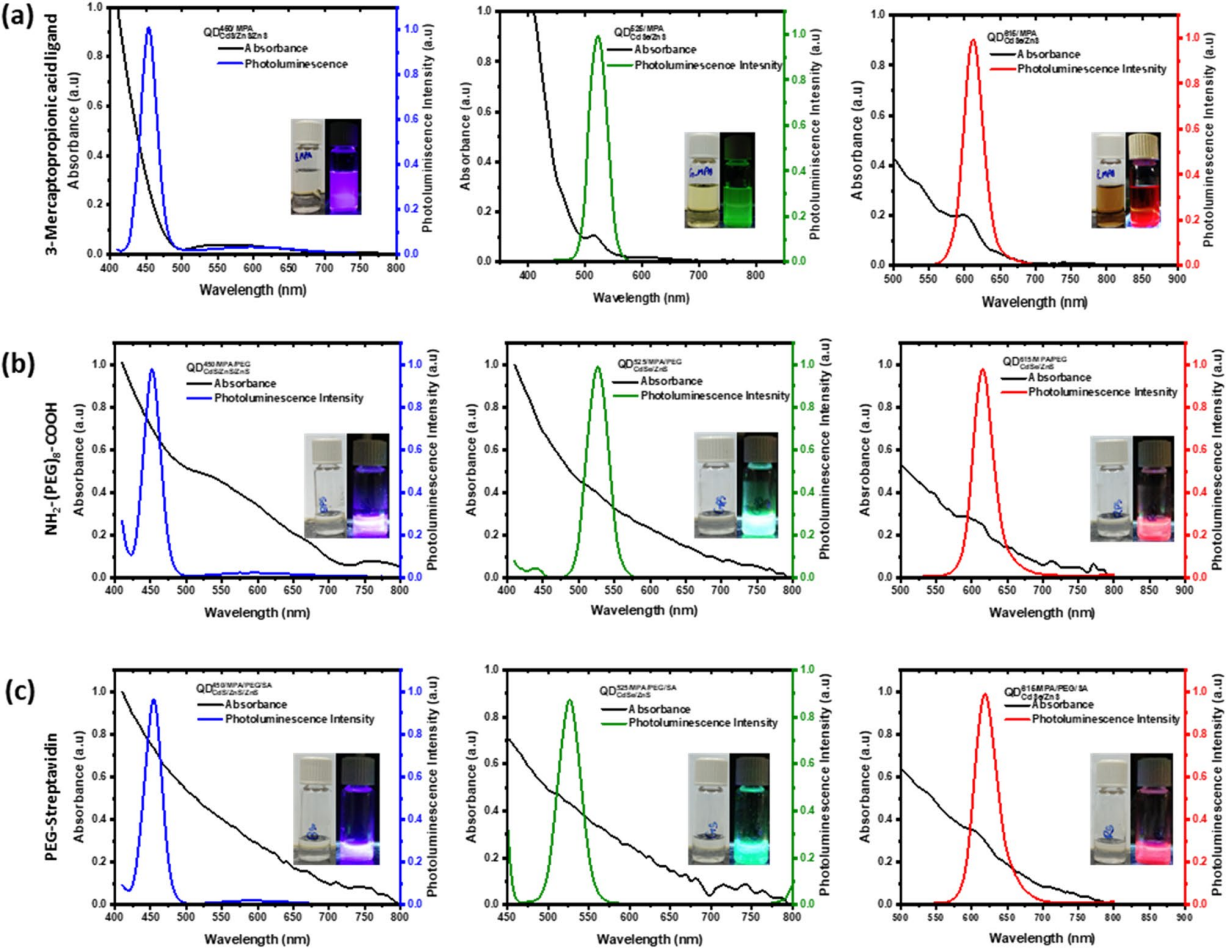
UV–Visible absorbance and fluorescence spectrum of Quantum dots (**a**) ${QD}_{CdS/ZnS}^{450/MPA}$, ${QD}_{CdSe/ZnS}^{525/MPA}$, and ${QD}_{CdSe/ZnS}^{615/MPA}$ quantum dots surface exchange with 3-Mercaptopropionic acid showing (**b**) ${QD}_{CdS/ZnS}^{450/MPA/PEG}$, ${QD}_{CdSe/ZnS}^{525/MPA/PEG}$, and ${QD}_{CdSe/ZnS}^{615/MPA/PEG}$ quantum dots surface conjugated with PEG (**c**) ${QD}_{CdS/ZnS}^{450/MPA/PEG/SA}$, ${QD}_{CdSe/ZnS}^{525/MPA/PEG/SA}$, and ${QD}_{CdSe/ZnS}^{615/MPA/PEG/SA}$ quantum dots surface conjugated with PEG and Streptavidin molecules showing the respective absorbance and fluorescence.

The UV–Visible AB and PL spectrum of ${QD}_{CdS/ZnS}^{450/MPA}$, ${QD}_{CdSe/ZnS}^{525/MPA}$, and ${QD}_{CdSe/ZnS}^{615/MPA}$ as shown in Fig. 4, the insight images show the consistency of the fluorescence after the ligand exchange. The dynamic light scattering (DLS), as shown in the supplementary information Fig S2, refers to the hydrodynamic diameter of respective QD ranges between 15 and 20 nm, which was appropriate for a core/shell diameter. Because of the hydrophilic nature of ligands and the distribution of carboxyl anchoring groups exhibit zeta potential, as shown in the supplementary information Fig. S3 with the net negative charge for MPA ligand, which are consistent with the previous reports^46,53^.

To observe the stability of the QD’s and for the biocompatible applications, we further assessed the QDs based on the fluorescence emissions for seven days. The respective QDs were allowed to incubate in the routine physiological solutions, including the 1X PBS, Millipore grade water, and DMEM (containing 10% FBS). The fluorescence intensity was slightly changed even after the QDs were incubated for seven days signify the stability, which are showed in the supplementary information Fig. S4. To assess the biocompatibility of the prepared QD’s (${QD}_{CdS/ZnS}^{450/MPA}$, ${QD}_{CdSe/ZnS}^{525/MPA}$, and ${QD}_{CdSe/ZnS}^{615/MPA}$.), MTT tests were performed on MCF-7, HeLa, HEK-293, and THP-1 cell lines which are incubated for 24 h. When the administered QD^MPA^ concentrations ranged from 0.1 to 100 µg/ml, the cell viability of the three QD’s groups remained over 90% at the 0.1 to 1.0 µg/ml as shown in the supplementary information Fig. S5. The cell survival after 24 h treatment reduced as the QD concentration rose, indicating that the MPA capped QD’s exhibited a concentration-dependent cytotoxicity pattern in cell viability.

### Antibody conjugation with quantum dots

To conjugate the biotinylated antibodies on the QDs, the above prepared QD^MPA^ were subject to modification with PEG to enhance the stability and shelf life of the QDs followed by conjugation with SA molecules. The PEG modification was achieved by using EDC/NHS coupling. The same EDC/NHS coupling was also used for the streptavidin molecules conjugation. The successful transformation of the PEG and SA were confirmed by the FTIR spectrum implicating the presence of native functional groups, including amine (−C=N) characteristic stretching bands at 1638–1646 cm^−1^ followed by appearance of strong bands at 1205–1250 cm^−1^ specific for the carboxyl (−C−O) groups. The existence of water molecules is indicated by the hydroxyl (-OH) group vibration peak at 3324–3352 cm^−1^ as shown in the supplementary information Fig S1. The surface functionalization for individual QD’s include ${QD}_{CdS/ZnS}^{450/MPA/PEG/SA}$, ${QD}_{CdSe/ZnS}^{525/MPA/PEG/SA}$, and ${QD}_{CdSe/ZnS}^{615/MPA/PEG/SA}$ were observed for the PL emission as shown in Fig. 4. The size of the respective QDs with PEG and SA are studied with DLS, the hydrodynamic size ranges between 20 and 40 nm were shown in the supplementary information Fig S2, the hydrophilic nature of PEG shows net negative charge, whereas SA showed net positive charge for the zeta potential measurements, which are shown in the supplementary information Fig S3.

To conjugate the QDs with antibodies, the concentration of the Abs were determined based on the UV–visible spectral data, where the absorbance at the peak maxima are incorporated in the theoretical calculations as mentioned in the Taniguchi and Green 2015 research reports, which are slightly reliable with the experimental data^54^. Antibodies include Anti-EpCAM, Anti-CD45, and Anti-CD44, which have biotin ends that are dispersed in 1X PBS at pH 7.2. These are allowed to couple with respective SA conjugated QDs in an equimolar 1:1 ratio, to maximize the coupling of the individual antibodies for specific cell line identification based on the previously reported protocols^55,56^. The QDs for antibodies for specific cell applications are shown in Table 1 and supplementary information Table S1. The relative efficacy of the QDs was further analyzed with the co-cultures .

**Table 1:** Quantum dots coupled with antibodies, based on the requirement of the targeted cells.

Quantum Dots	Antibodies	Conjugate	Targeted cells
${QD}_{CdS/ZnS}^{450/MPA/PEG/SA}$	Anti-EpCAM	${QD}_{CdS/ZnS}^{450/MPA/PEG/SA/EPCAM}$	Epithelial cells
${QD}_{CdS/ZnS}^{525/MPA/PEG/SA}$	Anti-CD45	${QD}_{CdS/ZnS}^{525/MPA/PEG/SA/CD45}$	Mesenchymal cells
${QD}_{CdS/ZnS}^{615/MPA/PEG/SA}$	Anti-CD44	${QD}_{CdS/ZnS}^{615/MPA/PEG/SA/CD44}$	Epithelia cells

### Binding efficacy of the antibody conjugated quantum dots

To evaluate the binding efficacy of the QD^Ab^, we opted for the co-cultured cell lines which mimic the peripheral blood samples. The standard culture conditions are maintained for the cell lines optimal growth, including MCF-7, HeLa, HEK-293, with THP-1. The QDs for normalized fluorescence intensity counts for specific cell lines are shown in supplementary information Table S1 and Fig S6. After 24 h of cells incubation, aliquots of specific cell line groups are prepared, followed by incubation of the QDs with incremental concentrations from 0.0001 to 1 μg/mL. At higher concentrations of QDs, i.e., of each aliquot for the specific cell lines, where MCF-7 cell lines show higher EpCAM+ at 1 μg/mL with lower cytotoxicity as shown in the supplementary information Fig S8. While of HeLa and HEK-293 cells showed lower EpCAM+ , and THP-1 cells showed strong CD45+ in all respective co-cultures. For reference, all the cell lines except THP-1 (which are CD45+) are infested with CD44+ to represent the efficacy of the QD in the co-cultures to define the heterogeneity of the cells in our present study .

### Optimization of the antibody conjugated quantum dots

**Figure 5:**
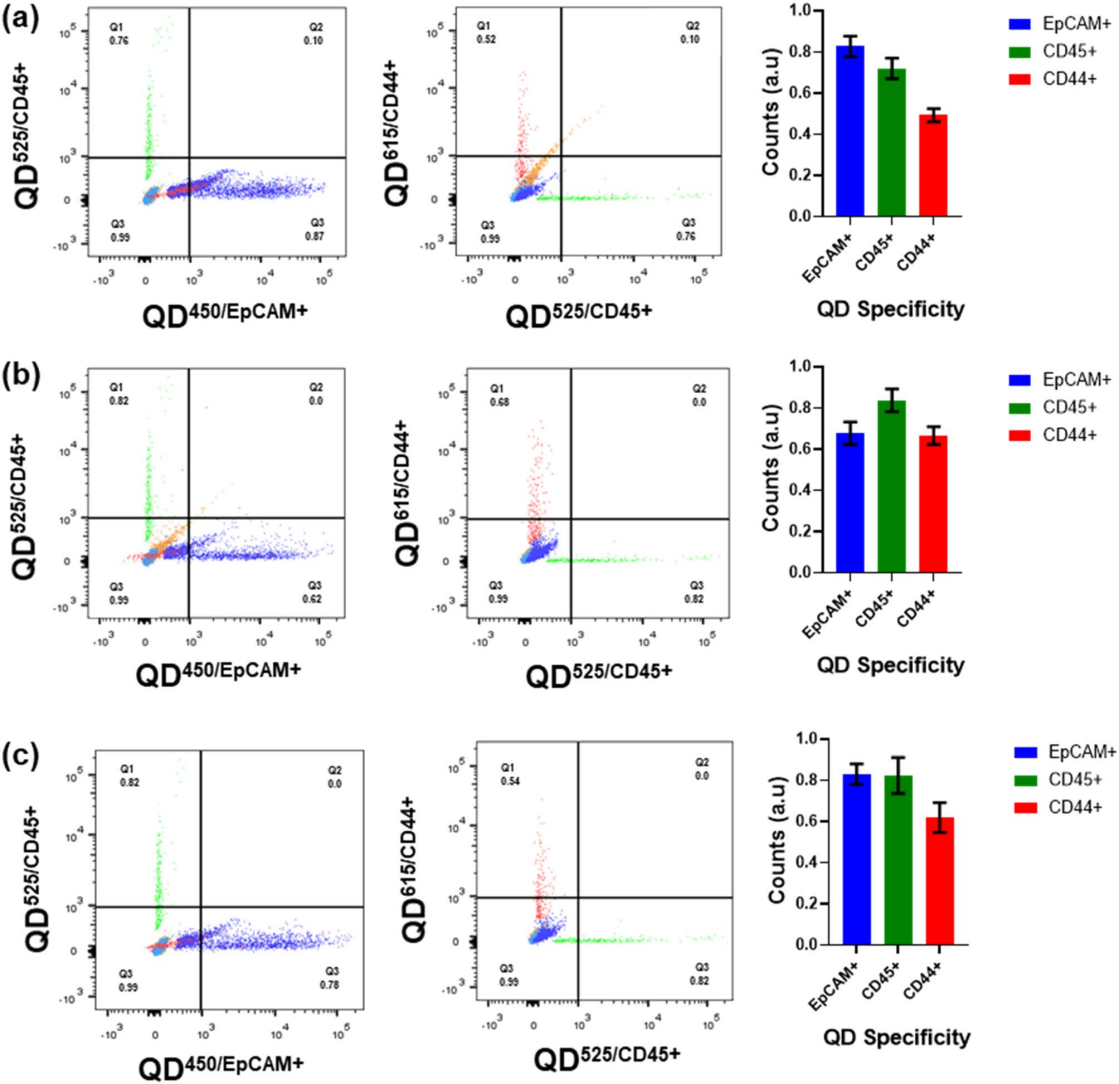
Flowcytometry graphical images for capture the EpCAM+ and EpCAM− cells by using the quantum dot conjugated with anti-EpCAM, anti-CD45 and anti-CD44 cells in co-culture cells in in-vitro incubated with concentrations at 1.0 μg/mL in 1 × PBS media were analyzed through flowcytometry (**a**) MCF-7 and THP-1 (**b**) HELA and THP-1 (**c**) HEK-293 and THP-1 cancer cell lines co-cultures with respective fluorescence emission and insight histograms shows the flowcytometry with respective Quantum dots binding. (The data is normalized to 1.0 which is equivalent to 100%, the data should be compared based on the relative normalized to percentages).

**Figure 6:**
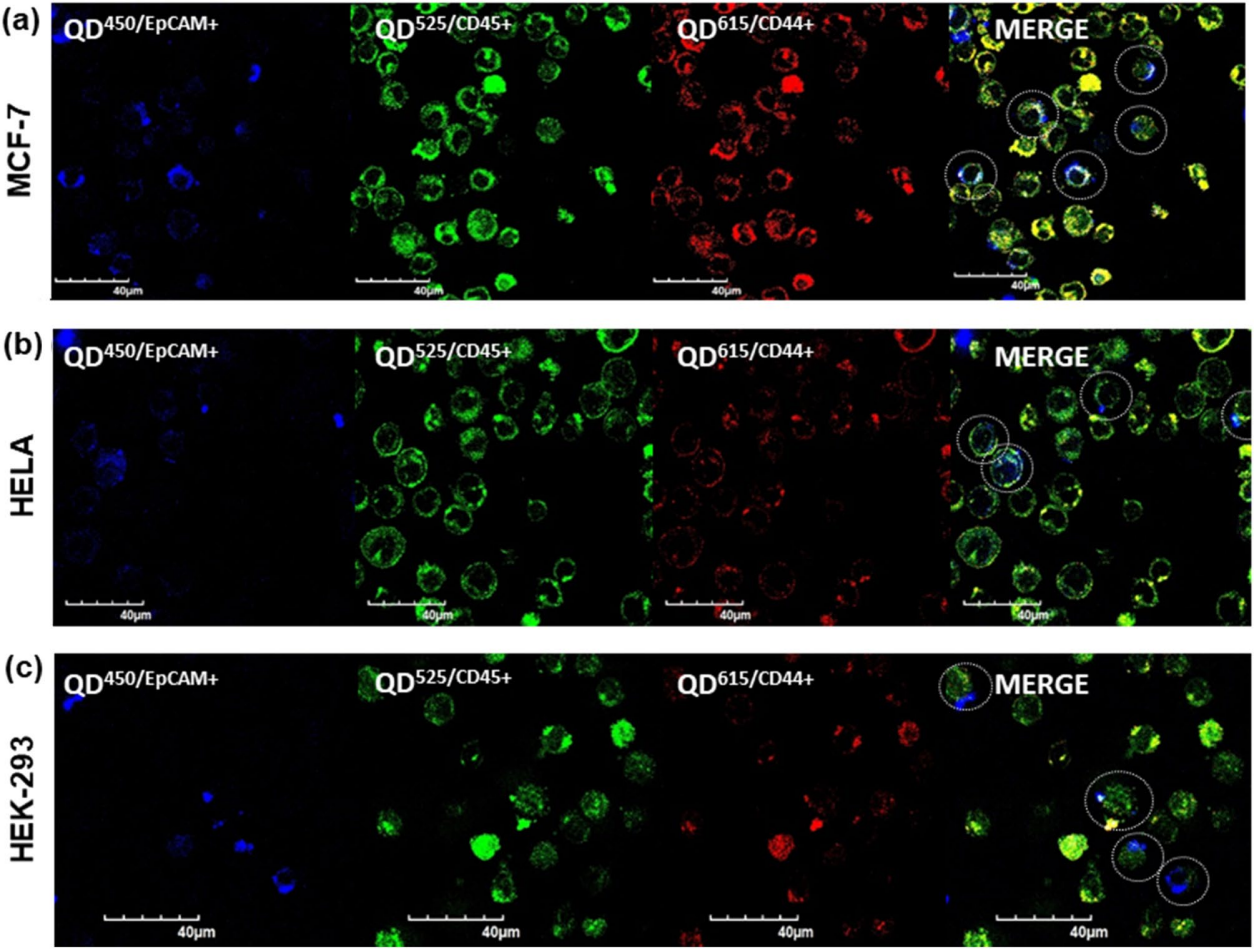
Confocal microscopic images for capture the EpCAM+ and EpCAM− cells by using the quantum dot conjugated with anti-EpCAM, anti-CD45, and anti-CD44 cells in co-culture cells in in-vitro incubated with concentrations at 1.0 μg/mL in 1 × PBS media were analyzed through confocal imaging (**a**) MCF-7 and THP-1 (**b**) HeLa and THP-1 (**c**) HEK-293 and THP-1 cancer cell lines co-cultures with fluorescence emissions shows the confocal images with respective Quantum dots binding, which are represented with dotted circles.

Aliquots of 5 ml containing co-cultures of HeLa, MCF-7, and HEK-293 with THP-1 cells as shown in the supplementary information Table S2 are allowed to grow for 24 h under optimal conditions and are observed for 60–80% confluency. The set of prelabeled vials with MCF-7, HeLa, and HEK-293 are co-cultured with THP-1 cell lines are pre-stained with the QD’s (${QD}_{CdS/ZnS}^{450/MPA/PEG/SA/EPCAM}$, ${QD}_{CdS/ZnS}^{525/MPA/PEG/SA/CD45}$, ${QD}_{CdS/ZnS}^{615/MPA/PEG/SA/CD44}$). These are incubated for 15 min, followed by 1 × PBS washes, and stored at lower temperatures (4–8 °C) before being subjected for sorting through flow cytometry. Using the standard protocol, the prelabeled vials containing cell lines are allowed to sort based on the fluorescence color intensities. The sorted cells were collected using sterile vials and were stored at freezing conditions for further imaging. The flow cytometry results as shown in Fig. 5 (refer to supplementary information Fig S7 for related raw data), revealed the contribution of the heterogeneity in the given in-vitro sample. The percentage of MCF-7 cell lines are greater than 87%, which are more sensitive than the HeLa about 62% and HEK-293 cells about 78% with more efficacy for EpCAM+ and with reference to CD44+ about 70%, while THP-1 cell are about 80% for CD45+ . The histogram data in Fig. 5 represents the comparative heterogeneity of the MCF-7, HeLa and HEK-293 cells with EpCAM+ along with reference indicator CD44+ and EpCAM− cells with CD45+ for THP-1 cells. These flowcytometry based sorted cells are subjected to imaging through confocal laser microscopy; the sorted cells are observed with fine borders, implicating healthier cells even after a longer exposure with bright blue intensity for EpCAM+ , green for CD45+ and bright red color for CD44+ , which are referred to Fig. 6.

### The capture of EpCAM+ and EpCAM− cells

**Figure 7:**
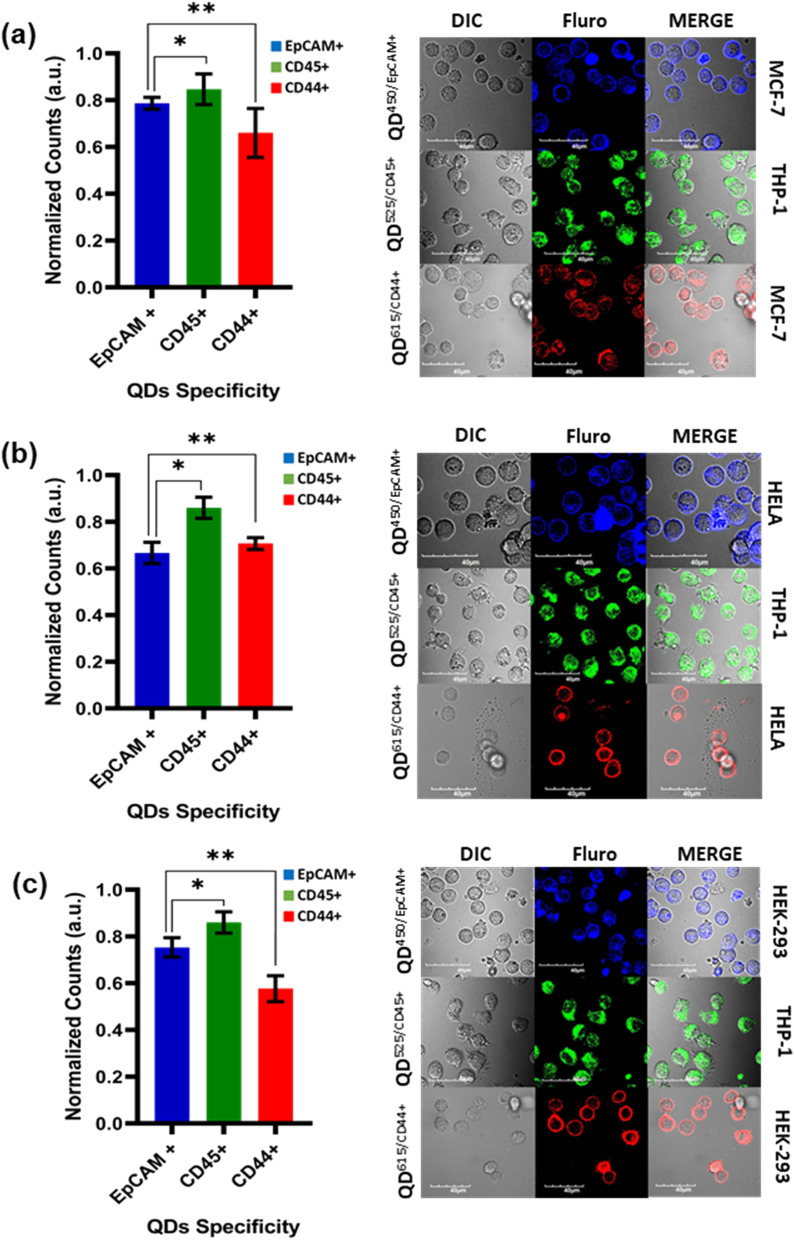
Multiple trails for each cell line in the co-cultures incubated with respective quantum dot antibody conjugates in-vitro with incubated with concentrations from 1.0 μg/mL media for significant analysis through Fluorescence-activated Cell Sorting and confocal imaging (**a**) MCF-7, and THP-1 (**b**) HeLa, and THP-1 (**c**) HEK-293, and THP-1 and HeLa cancer cell lines co-cultures with respective fluorescence emission shows the quantum dots binding. The statical data shows the probability of the occurrence of the EpCAM+ and EpCAM− cells, through reference through T-test was performed; “*” stands for the significance level < 0.001, and “**” stands for the significance level < 0.0001. (The data is normalized to 1.0 which is equivalent to 100%, the data should be compared based on the relative normalized to percentage).

Based on the optimized data from the QD’s binding efficacy, a series of trials were performed as designed, reaching to the last step of the protocol to capture EpCAM+ and EpCAM– (CD45+ and CD44+) cells. The detailed trials are shown in supplementary information Table S2 to represent the desired co-cultures. Each series are prelabeled for MCF-7, HeLa, HEK-293, and THP-1, which are co-cultured with optimal growth conditions for 24 h. The trail I assigned for the co-cultures of the MCF-7 and THP-1 cell lines showed a better affinity with 76% capture of EpCAM+ , CD45+ 78% and CD44+ 54%, which is shown in Fig. 7a. Trail II assigned for the co-cultures of the HeLa and THP-1 cell lines showed a better affinity with 62% capture of EpCAM+ , CD45+ 82% and CD44+ 68%, as shown in Fig. 7b. While trail III assigned for the co-cultures of the HEK-293 and THP-1 cell lines showed a better affinity with 72% capture of EpCAM+ , CD45+ 82% and CD44+ 54% as shown in Fig. 7c.

Furthermore, we investigated the efficacy of the concept of capturing the EpCAM+ and EpCAM− cell lines using QDs by multiple trials for each sample listed in Table 2. We could detect the EpCAM+ cells, despite showing fewer events and cross-checked with confocal imaging and flow cytometry sorting methods. The events for EpCAM+ were more effective for MCF-7 and HEK-293 than the HeLa cell line co-cultured with THP-1.

**Table 2:** Cells captured when treated with respective Antibody Quantum dots Conjugates.

QD^Ab^ Conjugates	Cells Sorted (%)		
	MCF-7	HELA	HEK-293
EpCAM+	9.45 ± 0.8	7.71 ± 0.9	8.84 ± 0.2
CD45+	3.76 ± 0.6	2.97 ± 1.0	4.77 ± 0.5
CD44+	3.25 ± 0.6	1.60 ± 1.0	2.03 ± 0.7
EpCAM/CD44+	4.79 ± 0.8	6.07 ± 0.3	6.21 ± 0.4
EpCAM/CD45+	2.86 ± 0.4	2.60 ± 0.5	3.13 ± 0.9
CD45/CD44+	1.50 ± 1.0	1.31 ± 0.9	1.62 ± 0.9

## Discussion

We successfully synthesized the bright and stable quantum dots with three color fluorescence emissions from blue, green and red when excited with 405 nm laser. We demonstrated the synthesis of the QDs soluble in aqueous solvents such as Millipore grade water and have lower cytotoxicity, enabling them to be biocompatible for cellular imaging and sorting experiments. Further, we also showed the consistency in the fluorescence emissions during all the surface modifications from sequential addition of MPA, PEG and SA to QD’s. An active SA molecule on the surface of QD’s provides a strong interaction with the biotinylated antibodies such as Anti-EpCAM, Anti-CD45 and Anti-CD44 to prepare the QD^Ab^.

In our current study, the primary objective was to investigate the occurrence of rare cancer cells based on the specific binding of QD^Ab^ for the EpCAM+ and EpCAM− (CD44+ and CD45 +) cell lines in-vitro based peripheral blood mimic sample co-culture models. The goal was achieved successfully by showing the EpCAM+ cells in MCF-7, HeLa and HEK-293, whereas EpCAM− for THP-1 cell lines. Our cell line identification method enables us to provide the real-time assessment of the heterogeneity of the rare cancer cells in given in-vitro sample, which offers rapid detections of the EpCAM+ and EpCAM− cells. Our present study had shown the possible disease progression through heterogenic transformation of the cells in a co-culture system to mimic the metastatic phases. A few events of morphological heterogenic transition were observed, which are similar in certain transitions detected based on the Abs binding to the cell such as EMTs, which are noted from earlier reports and considered to be EpCAM/CD44+^57^ and EpCAM/CD45+^58^. Our approach suggests the existence of more number CD44+ cells, which is a well-known marker for EMT groups^59,60^. Whereas, some of the cells are positive for CD44/CD45+ , which are more efficient in the metastasis EMT and MET groups^18,33^.

We successfully demonstrate the in-vitro heterogeneity of the cancer cells when subjected to QD^Ab^ with specific concentrations. Basing on the standard plot the capture efficacy was determined using different concentration of the QDs bound on the surface of the cells, followed by optimization for cellular sorting based on the respective fluorescence intensities emissions through FACS (Fluorescence-activated cell sorting) as shown in Fig. 5. Identification of the rare cancer cell events in the current investigation is confined to EpCAM+ /CD44+ (Epithelial cells)^57,61^ and CD45+ (Mesenchymal cells)^58,62^. Given these constraints, we would expect QD^450/EPCAM^ staining to signify for EpCAM+ cells. These cells are familiar with the CTCs due to the known fact from the earlier reports that the CTCs are EpCAM+ in the case of epithelial cancers^39,42,63^.

The QD^450/EPCAM^ cells were added with EMT markers (QD^615/CD44^) because EpCAM+ cells are escaping tumor cells derived from primary tumor tissue and are most likely to be mesenchymal EpCAM+ cells. Given the link between EpCAM+ cells and recurrence in this study, we hypothesized that the QD’s platform with staining for mesenchymal cancer markers (e.g., CD45/EpCAM and CD44/EpCAM) would be capable of more precise detection of rare heterogenic cells, via in-vitro multiplex staining for distinguishing epithelial and mesenchymal cancers. There was a significant difference in the EpCAM+ and EpCAM− groups in our sample (*p* = 0.001, and *p* = 0.0001), as shown in Fig. 7. However, we obtained meaningful data for the difference between the CCH groups by analyzing the type of cancer cells and cultures recurrence.

The EpCAM+ cells were related to the systemic recurrence pattern in co-cultures, along with CD45+ , CD44+ , EpCAM/CD45+ , EpCAM/CD44+ , and CD45/CD44+ with overall sub-populations a shown in Table 2. These may be connected to the limitations of our investigation, which included a small number of cells and a short period of follow-ups. To raise the clinical value of EpCAM+ cancer cells and validate the diagnostic capability of CCH, one of the crucial components is the use of several antibodies to boost sensitivity and reduce CCH loss, followed by larger-scale research over a longer period. As the CCH is typically shed from tumor tissue, it is critical to examine the EMT/MET cancer stem cell markers status for EpCAM+ in addition to tumor location. These findings will contribute to CCH research in various cancer investigations and therapeutics.

## Conclusions

Our results demonstrate the availability of an efficient method for analyzing metastasis progression by identifying rare cancer cell heterogenic events in an in-vitro co-culture model. With more precision and accuracy, the current defined protocol successfully assessed rare cancer cell events based on the EpCAM+ and EpCAM− marker systems. The results generated were cross-checked with similar trails with different combinations for MCF-7, HeLa, HEK-293, and THP-1 cell lines. This principal method may help to deliver a new diagnostic approach in identifying the early prognosis, metastatic progression of the disease and can be most promising investigation for targeted therapy with a simple in*-vitro* co-culture model.

## Supplementary Information

Supplementary Information.
