# Supplementary material for: Quantum dots based in-vitro co-culture cancer model for identification of rare cancer cell heterogeneity
Source: Sci Rep. 2022 Apr 7;12:5868. doi: 10.1038/s41598-022-09702-y (PMC8991261; doi:10.1038/s41598-022-09702-y)
Supplement: Supplementary file 1 — Supplementary Information. [file 41598_2022_9702_MOESM1_ESM.docx]

**Supplementary Information**

**Quantum dots based *in-vitro* co-culture cancer model for identification of rare cancer cell heterogeneity**

Satyanarayana Swamy Vyshnava^1^, Gayathri Pandluru^1^, Kanderi Dileep Kumar^2^, Shiva Prasad Panjala^3^, Swathi Banapuram^3^, Kameshpandian Paramasivam^4^, Kothamunireddy Varalakshmi Devi^5^, Roja Rani Anupalli^3^ and Muralidhara Rao Dowlatabad*^1^

^1^Department of Biotechnology, University College of Sciences, Sri Krishnadevaraya University, Anantapuramu-515003, Andhra Pradesh, India.

^2^Department of Microbiology, University College of Sciences, Sri Krishnadevaraya University, Anantapuramu-515003, Andhra Pradesh, India.

^3^Department of Genetics, University College of Sciences, Osmania University, Hyderabad-500007, Telangana, India.

^4^Department of Forensic science, Ultra arts and science college, Madurai-625104, Tamil Nadu, India.

^5^College of Pharmaceutical Sciences, Sri Krishnadevaraya University, Anantapuramu-515003, Andhra Pradesh, India.

***Corresponding author**

Dr. Muralidhara Rao Dowlatabad,

Department of Biotechnology,

University College of Sciences,

Sri Krishnadevaraya University,

Anantapuramu, A.P, India. 515 003,

Email id: [rao.muralidhara@gmail.com](mailto:rao.muralidhara@gmail.com)


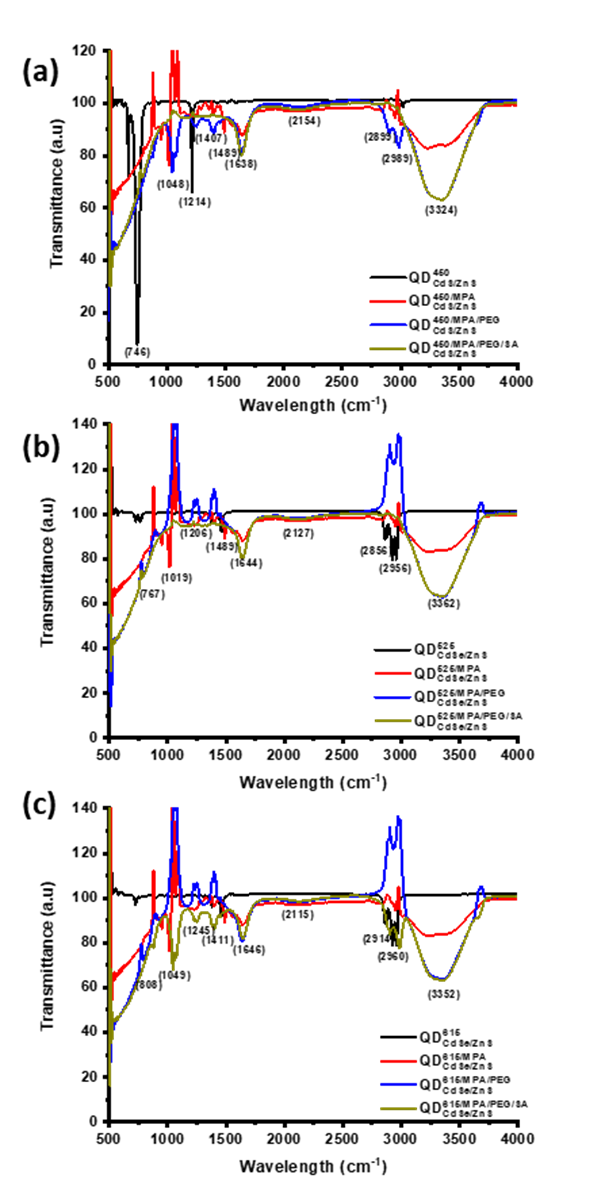


**Fig S1.** Fourier transform infrared spectroscopy of Quantum dots with 3-Mercaptopropionic acid followed by conjugated with NH_2_-(PEG)_8_-COOH and Streptavidin moieties (a) QD^450^ (b) QD^525^ (c) QD^615^


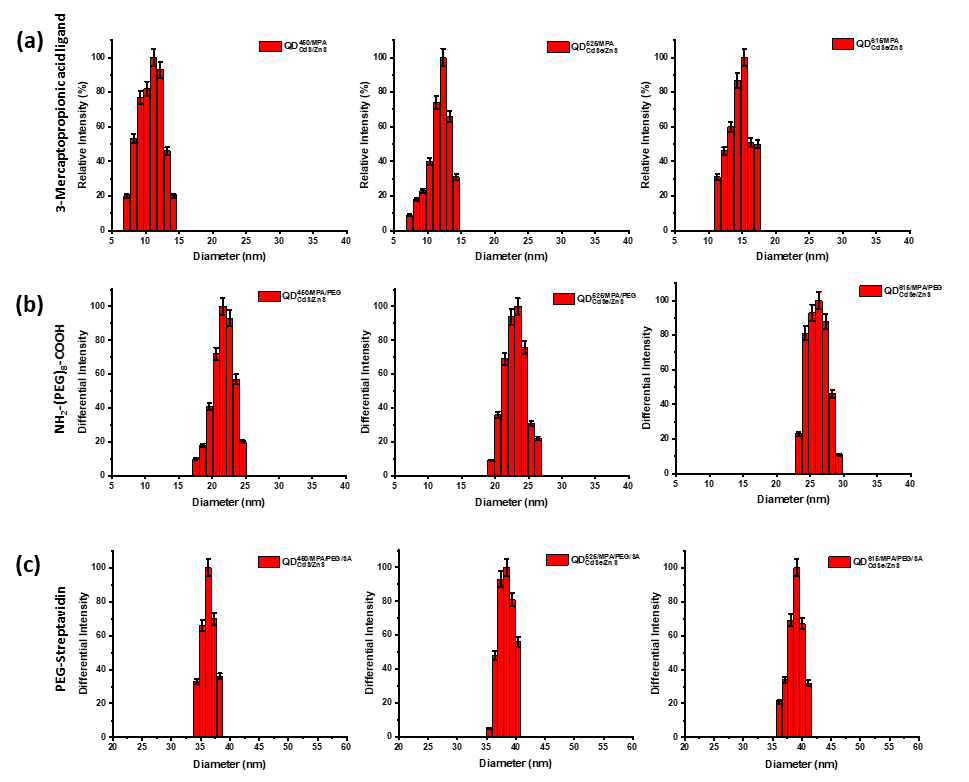


**Fig S2.** Dynamic light scattering of Quantum dots with 3-Mercaptopropionic acid followed by conjugated with NH_2_-(PEG)_8_-COOH and Streptavidin moieties showing the hydrodynamic sizes of (a) QD^450^ (b) QD^525^ (c) QD^615^


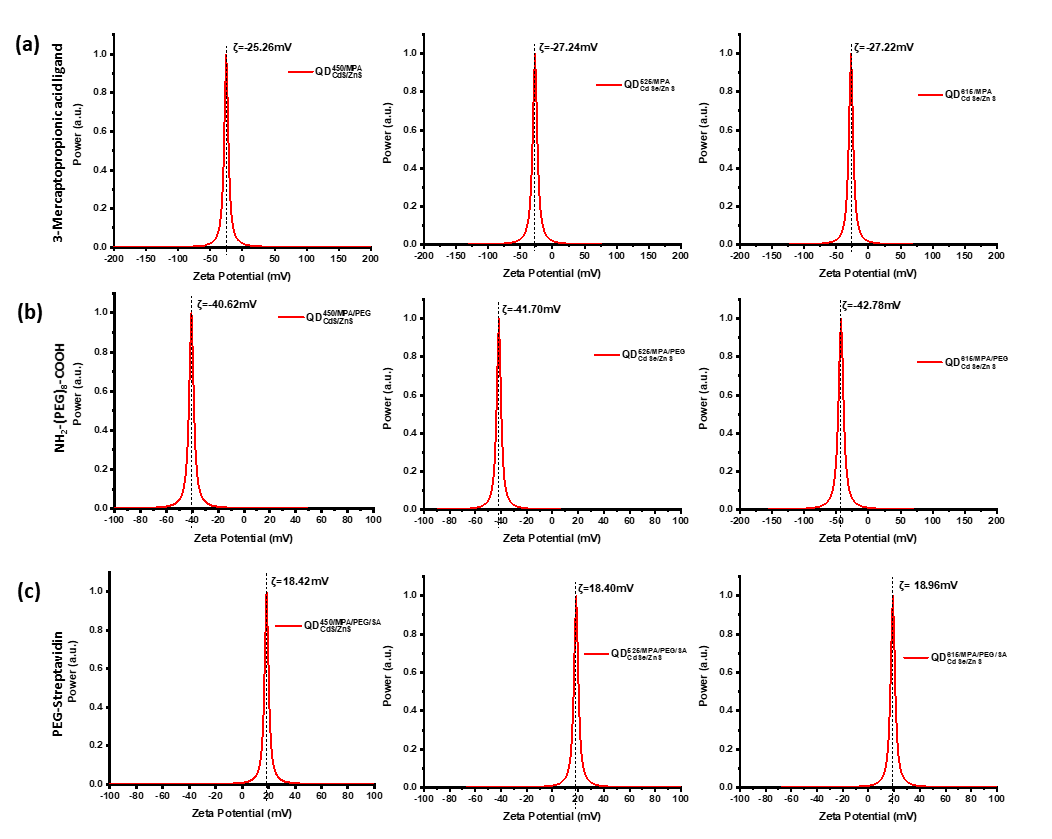


**Fig S3.** Dynamic light scattering- Zeta potential of Quantum dots with 3-Mercaptopropionic acid followed by conjugated with NH_2_-(PEG)_8_-COOH and Streptavidin moieties showing the hydrodynamic sizes of (a) QD^450^ (b) QD^525^ (c) QD^615^


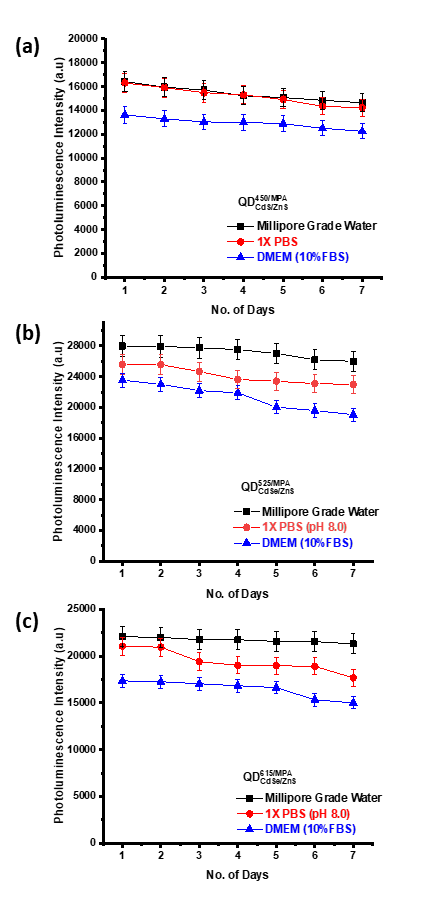


**Fig S4.** Stability of the Quantum dots studied based on the fluorescence intensities for a period of seven days which are dispersed in the Millipore grade water, 1X PBS and DMEM (10% FBS) (a) ${QD}_{CdS/ZnS}^{450/MPA}$ (b) ${QD}_{CdSe/ZnS}^{525/MPA}$ (c) ${QD}_{CdSe/ZnS}^{615/MPA}$.


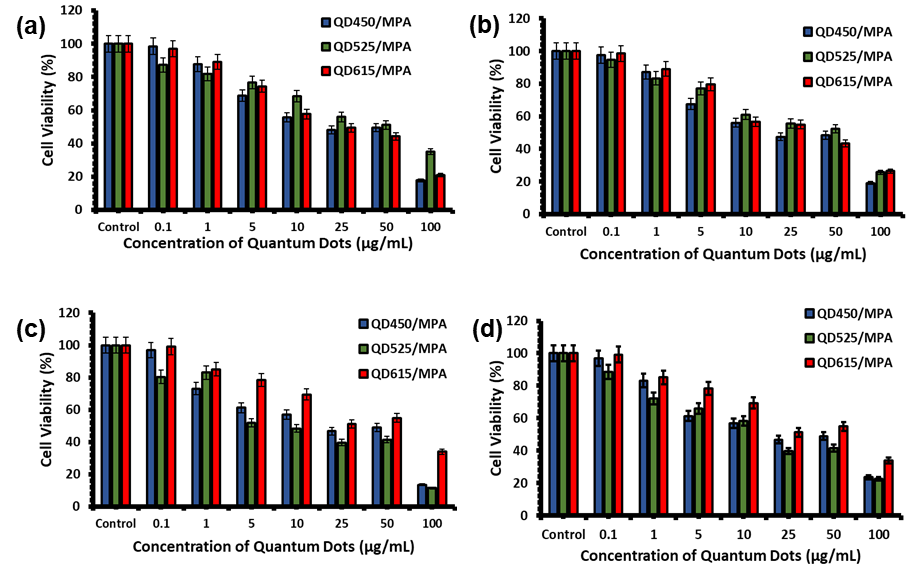


**Fig S5.** Cytotoxicity of the quantum dots incubated with concentrations from 0.1, 1.0, 5.0,10.0, 25.0, 50, and 100 μg/mL in DMEM media incubated for 24 hours **(a)** MCF-7 cytotoxicity was initiated at 5 μg/mL **(b)** HeLa cytotoxicity was initiated at 5 μg/mL **(c)** HEK-293 cytotoxicity was initiated at 5 μg/mL **(d)** THP-1 cytotoxicity was initiated at 5 μg/mL, the working concentrations of these quantum dots will be at 1 μg/mL

**
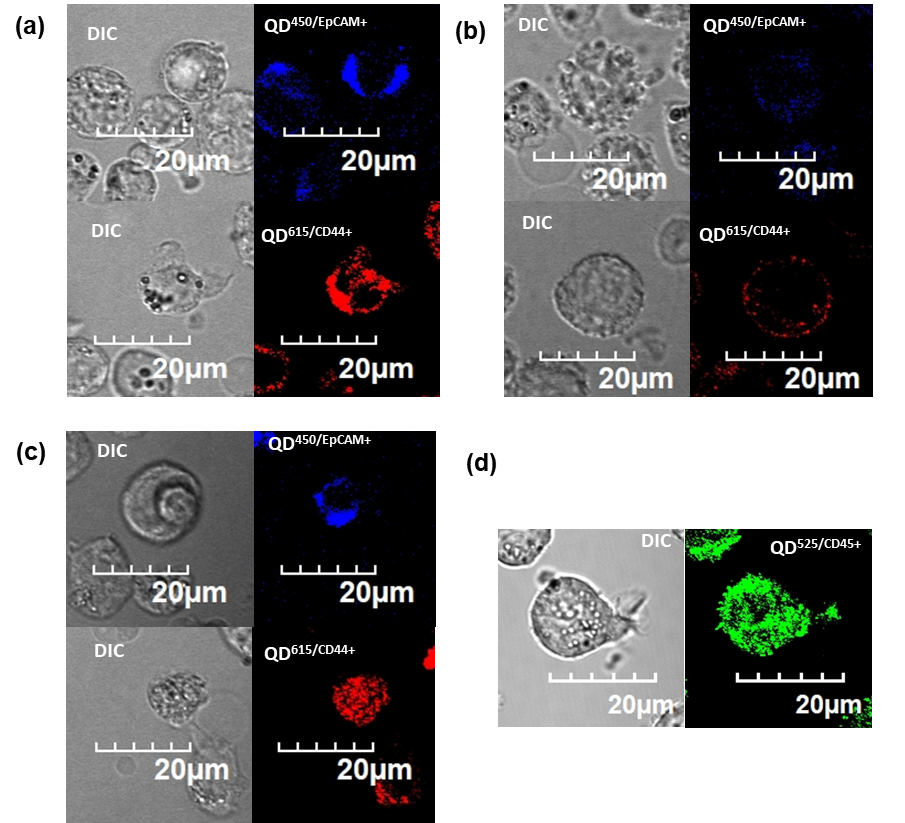
**

**Fig S6.** Confocal Microscopy imaging of the quantum dots conjugated with anti-EpCAM, anti-CD45 and anti-CD44 antibodies (a) MCF-7 cell lines with respective fluorescence emission for anti-EpCAM and anti-CD44 (b) HeLa cell lines with respective fluorescence emission for anti-EpCAM and anti-CD44 (c) HEK-293 cell lines with respective fluorescence emission for anti-EpCAM and anti-CD44 (d) THP-1 cell lines with respective fluorescence emission for anti-CD45


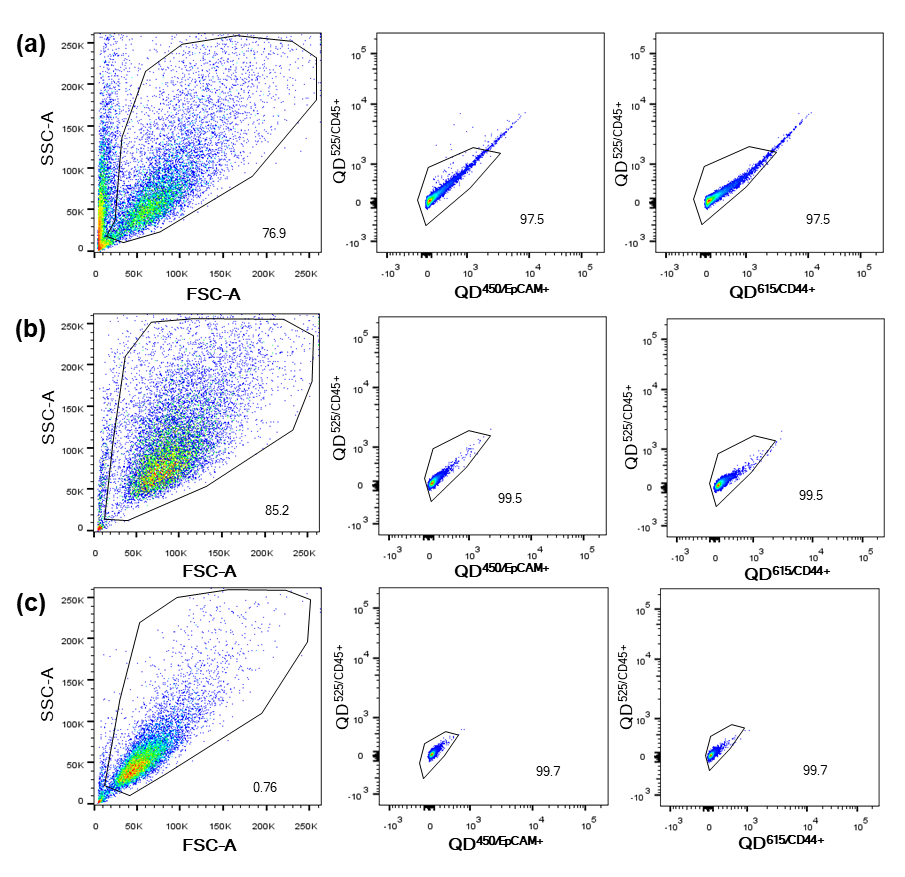


**Fig S7.** Fig S7. Flowcytometry dot plot images for controls, where the cells lines are in co-culture incubated in 1x PBS media were analyzed through flowcytometry (a) MCF-7 and THP-1 (b) HELA and THP-1 (c) HEK-293 and THP-1 cancer cell lines co-cultures with absence of fluorescence emissions and insight histograms shows the flowcytometry with respective cells devoid of Quantum dots binding

**
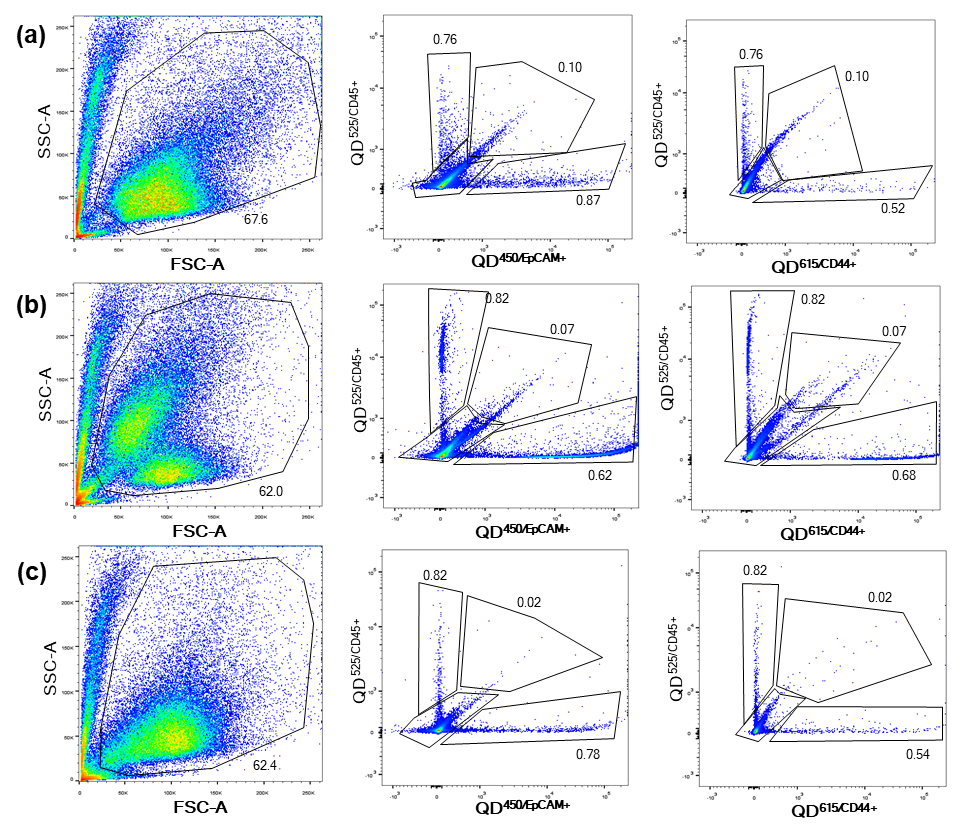
**

**Fig S8.** Flowcytometry dot plot images for capture the EpCAM+ and EpCAM- cells by using the quantum dot conjugated with anti-EpCAM, anti-CD45 and anti-CD44 cells in co-culture cells in in-vitro incubated with concentrations at 1.0 μg/5mL in 1x PBS media were analyzed through flowcytometry (a) MCF-7 and THP-1 (b) HELA and THP-1 (c) HEK-293 and THP-1 cancer cell lines co-cultures with respective fluorescence emission and insight histograms shows the flowcytometry with respective Quantum dots binding


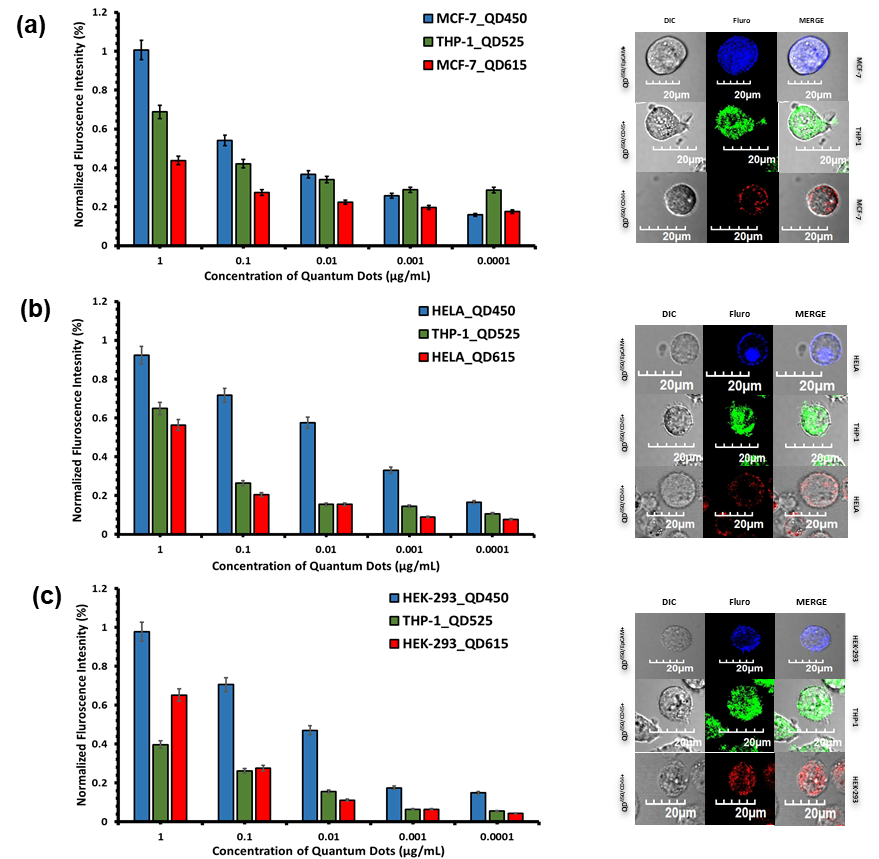
**Fig S9.** Binding efficacy of the quantum dots conjugated with anti-EpCAM, anti-CD45 and anti-CD44 cells in-vitro incubated with concentrations from 0.0001, 0.001, 0.01, 0.1 and 1.0 μg/mL in DMEM media incubated for 15 minutes (a) MCF-7 and THP-1 cancer cell lines co-cultures with higher fluorescence at 1.0 μg/mL concentration and insight shows the confocal images with respective Quantum dots binding (b) HeLa and THP-1 cancer cell lines co-cultures with higher fluorescence at 1.0 μg/mL concentration and insight shows the confocal images with respective Quantum dots binding (c) HEK-293 and THP-1 cancer cell lines co-cultures with higher fluorescence at 1.0 μg/mL concentration and insight shows the confocal images with respective Quantum dots binding

**
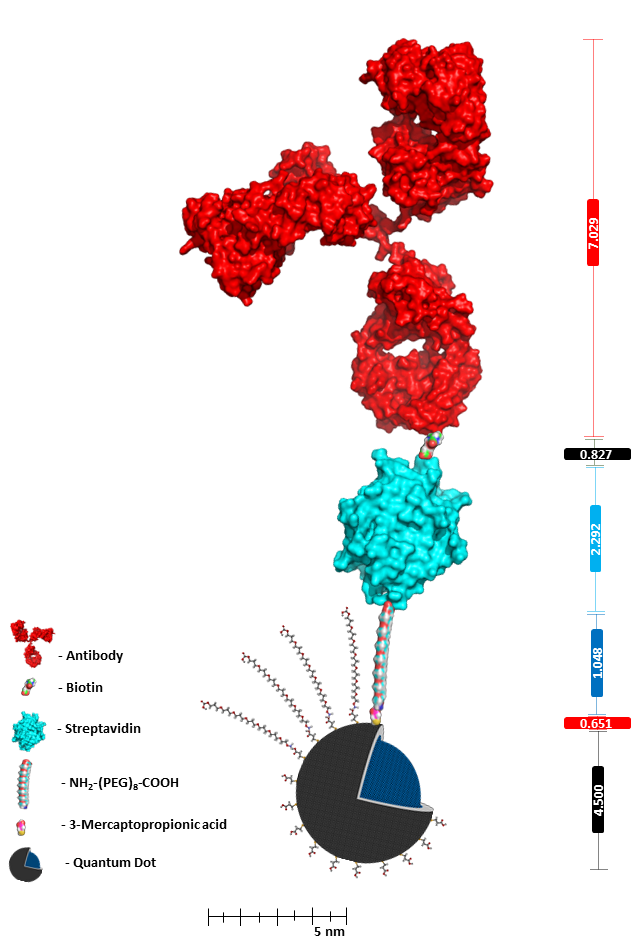
**

**Fig S10.** Structural illustrations of the functionalized Quantum dots **(Refer table S3 for details)**

**Table S1:** Quantum dots with specific fluorescence wavelength coupled with specific antibodies, based on the requirement of the targeted cell lines

| **Quantum dots** | **Fluorescence wavelength (nm)** | **Antibodies bind to QD** | **Cell lines** | **Cell count**  **No of cells/mL** |
| --- | --- | --- | --- | --- |
| **QD^450^** | 450 | EpCAM | MCF-7  HeLa  HEK-298 | 1x10^6^ |
| **QD^525^** | 525 | CD45 | THP-1 | 1x10^6^ |
| **QD^615^** | 615 | CD44 | MCF-7  HeLa  HEK-298 | 1x10^6^ |

**Table S2:** Aliquots of specific cell line groups for Quantum dots binding studies

| **Trails** | **Cell lines** | **QD specificity** | **Number of cells seeded for co-cultures** | **Mean cell count percentage sorted from flow cytometry** |
| --- | --- | --- | --- | --- |
| **I** | MCF-7 | **QD^450/EpCAM^** | 2x10^3^ | 76 |
|  |  | **QD^615/CD44^** | 2x10^3^ | 54 |
|  | THP-1 | **QD^525/CD45^** | 6x10^3^ | 78 |
| **II** | HeLa | **QD^450/EpCAM^** | 2x10^3^ | 62 |
|  |  | **QD^615/CD44^** | 2x10^3^ | 68 |
|  | THP-1 | **QD^525/CD45^** | 6x10^-3^ | 82 |
| **III** | HEK-293 | **QD^450/EpCAM^** | 2x10^3^ | 72 |
|  |  | **QD^615/CD44^** | 2x10^3^ | 54 |
|  | THP-1 | **QD^525/CD45^** | 6x10^3^ | 82 |

**Table S3:** Structural constituents of the Quantum dots

| **Molecule name** | **Molecular Weight**  **(g/mol)** | **Mass**  **(g/particle)** | **Density**  **(g/cm^3^)** | **Volume**  **(nm^2^/particle)** | **Spherical diameter**  **(nm)** |
| --- | --- | --- | --- | --- | --- |
| **3-Mercaptopropoinc acid** | 106.15 | 1.763x10^-22^ | 1.22 | 0.145 | 0.651 |
| **NH2-(PEG)8-COOH** | 496.55 | 8.245x10^-22^ | 1.37 | 0.602 | 1.048 |
| **Streptavidin** | 5200.00 | 8.635x10^-21^ | 1.37 | 6.303 | 2.292 |
| **Biotin** | 244.31 | 4.057x10^-22^ | 1.37 | 0.296 | 0.827 |
| **Antibody** | 150000 | 2.491x10^-19^ | 1.37 | 181.82 | 7.029 |
| **Biotin-Antibody** | 150244.31 | 2.496x10^-19^ | 1.37 | 182.12 | 7.856 |
